# Normal breast tissue DNA methylation differences at regulatory elements are associated with the cancer risk factor age

**DOI:** 10.1186/s13058-017-0873-y

**Published:** 2017-07-10

**Authors:** Kevin C. Johnson, E. Andres Houseman, Jessica E. King, Brock C. Christensen

**Affiliations:** 1 Department of Epidemiology, Geisel School of Medicine at Dartmouth, Lebanon, NH 03756 USA; 2 Department of Molecular and Systems Biology, Geisel School of Medicine at Dartmouth, Lebanon, NH 03756 USA; 3 The Jackson Laboratory for Genomic Medicine, Farmington, CT 06032 USA; 4 Department of Biostatistics, College of Public Health and Human Sciences, Oregon State University, Corvallis, OR 97331 USA; 5 Department of Community and Family Medicine, Geisel School of Medicine at Dartmouth, Lebanon, NH 03756 USA

**Keywords:** Normal breast, DNA methylation, Aging, 5mC, Risk factors, Reference-free, Breast cancer, Epigenetics, Epigenetic drift

## Abstract

**Background:**

The underlying biological mechanisms through which epidemiologically defined breast cancer risk factors contribute to disease risk remain poorly understood. Identification of the molecular changes associated with cancer risk factors in normal tissues may aid in determining the earliest events of carcinogenesis and informing cancer prevention strategies.

**Methods:**

Here we investigated the impact cancer risk factors have on the normal breast epigenome by analyzing DNA methylation genome-wide (Infinium 450 K array) in cancer-free women from the Susan G. Komen Tissue Bank (*n* = 100). We tested the relation of established breast cancer risk factors, age, body mass index, parity, and family history of disease, with DNA methylation adjusting for potential variation in cell-type proportions.

**Results:**

We identified 787 cytosine-guanine dinucleotide (CpG) sites that demonstrated significant associations (*Q* value <0.01) with subject age. Notably, DNA methylation was not strongly associated with the other evaluated breast cancer risk factors. Age-related DNA methylation changes are primarily increases in methylation enriched at breast epithelial cell enhancer regions (*P* = 7.1E-20), and binding sites of chromatin remodelers (MYC and CTCF). We validated the age-related associations in two independent populations, using normal breast tissue samples (*n* = 18) and samples of normal tissue adjacent to tumor tissue (*n* = 97). The genomic regions classified as age-related were more likely to be regions altered in both pre-invasive (*n* = 40, *P* = 3.0E-03) and invasive breast tumors (*n* = 731, *P* = 1.1E-13).

**Conclusions:**

DNA methylation changes with age occur at regulatory regions, and are further exacerbated in cancer, suggesting that age influences breast cancer risk in part through its contribution to epigenetic dysregulation in normal breast tissue.

**Electronic supplementary material:**

The online version of this article (doi:10.1186/s13058-017-0873-y) contains supplementary material, which is available to authorized users.

## Background

An effective way to decrease breast cancer-related morbidity and mortality is to identify individuals who may be at increased risk of developing breast cancer and apply early intervention strategies. In addition to inherited gene mutations, there are several demographic factors that are associated with an increased risk of breast cancer including increasing age, being overweight after menopause, alcohol intake, having never been pregnant (that is, nulliparous), earlier age at menarche, and a family history of breast cancer [1–4]. However, the underlying biologic mechanism(s) through which many of these epidemiologically defined breast cancer risk factors contribute to carcinogenesis remains unclear.

Biomarkers strongly associated with breast cancer risk factors provide an opportunity to understand cancer development. One such potential biomarker investigated for its role in the early detection of breast cancer is DNA methylation. DNA methylation is the covalent addition of a methyl group to cytosine, often in the context of a cytosine followed by a guanine in the 5' to 3' direction (that is, a cytosine-guanine dinucleotide (CpG)), and is necessary for cell-type-specific differentiation, including in the mammary gland [5, 6]. DNA methylation is a stable, yet modifiable epigenetic modification and DNA methylation alterations are known to occur early in breast carcinogenesis [7, 8]. It has been hypothesized that disease risk factors may mediate their disease-predisposing effects through perturbation of epigenomic control. Candidate gene studies in normal breast tissues indicate that DNA methylation changes are related to age and to other known breast cancer risk factors. For example, women without breast cancer, but at high risk (Gail model score) are more likely to have aberrant methylation of the tumor suppressor genes *APC* and *RASSF1* compared with women at low risk [9]. In another candidate gene study of normal breast tissue, the same group observed that *RASSF1* methylation is associated with breast cancer risk level, and that increasing parity is associated with decreased *APC* methylation [10]. More recently, a study identified cancer-related field defects in DNA methylation based on study of both normal breast tissues from disease-free subjects and tumor-adjacent normal breast tissues [11]. In addition, preliminary results from another study provide evidence that genome-wide age-related DNA methylation changes in tumor-adjacent normal breast tissues are more likely to be altered in breast tumors than in randomly selected regions [12]. However, the relationship between breast cancer risk factors and DNA methylation changes in normal breast tissue from disease-free subjects remains unclear.

Here we extended the foundational work to tissues from disease-free women with detailed breast cancer risk factor data and applied more comprehensive epigenomic profiling methods. We tested the relationship between DNA methylation patterns and breast cancer risk factors such as age, body mass index (BMI), and reproductive and family history, using an epigenome-wide association study (EWAS) approach. Importantly, we adjusted for potential variation in cellular proportions across samples. Age is the strongest risk factor for breast cancer and we have shown that the patterns of age-related DNA methylation are dependent upon genomic context and that these age-related methylation patterns were consistent across normal breast tissue from independent populations. We found that these molecular alterations become further altered in pre-invasive and invasive cancerous lesions. Together, the epigenetic changes we identified here provide insights into how the breast cancer risk factor of age may influence disease development.

## Methods

### Study population

The discovery population consisted of 100 cancer-free women who donated breast tissue biopsy specimens to the Susan G. Komen Tissue Bank after providing written informed consent. We selected biospecimens from women with a biopsy that scored for a high proportion of epithelial cells as determined by the Susan G. Komen Tissue Bank study pathologist (*n* = 100) [13]. The sample population was selected for an approximately equal distribution of parous and nulliparous women, and to include a wide age range of subjects. Subject demographic and breast cancer risk factors were collected from tissue donors using a questionnaire administered by the Susan G. Komen Tissue Bank. Family history of cancer was defined by whether or not the donor had at least one first-degree blood relative (i.e., mother or sister) diagnosed with breast cancer. This work was performed in accordance with the ethical principles in the Declaration of Helsinki.

### DNA methylation quantification and normalization

Fresh-frozen tissue samples were manually dissected and DNA was extracted using Qiagen DNeasy Blood and Tissue Kit according to the manufacturer’s protocol (Qiagen, Valencia, CA, USA). DNA was quantified using a Qubit fluorometer and 1 ug of DNA was then bisulfite-modified using the EZ DNA methylation kit (Zymo Research, Orange, CA, USA) according to the manufacturer’s recommended protocol. The resulting material was used as input for the hybridization on the Infinium HumanMethylation450 BeadChip (Illumina, San Diego, CA, USA). Samples were randomized to plates and subjected to epigenome-wide DNA methylation assessment. The methylation status for each CpG locus was calculated as the ratio of fluorescent signals (β = Max (M, 0)/[Max(M,0) + Max(U,0) + 100]), ranging from 0 (non-methylated) to 1 (completely methylated), using average probe intensity for the methylated (M) and unmethylated (U) alleles. Normalization and background correction of raw signals was performed using the *FunNorm* procedure available in the R/Bioconductor package *minfi* (version 1.10.2) [11]. Illumina probe-type normalization was carried out with beta-mixture quantile normalization (BMIQ) [14]. Prior to analysis we removed CpG sites on sex chromosomes, and those corresponding to probes previously identified as cross-reactive or containing single nucleotide polymorphisms (SNPs), resulting in 390,292 CpGs remaining for analysis [15].

### Validation in independent populations and The Cancer Genome Atlas

Independent breast tissue samples were available from the National Disease Research Interchange (NDRI, GSE74214, *n* = 18) and The Cancer Genome Atlas Database (TCGA, *n* = 97) [16]. Raw intensity data (IDAT) files were available for both studies and DNA methylation data were processed and normalized using the same methods described above. Likewise, raw DNA methylation IDAT files were accessed and processed using the same methods outlined above for both ductal carcinoma in situ (*n* = 55, GSE66313) and invasive ductal carcinoma (*n* = 749, TCGA) to compare DNA methylation differences between normal-adjacent tissue and pre-invasive or invasive lesions [8].

### Statistical analysis

All data analysis was conducted in R version 3.3.1.

#### Cell-mixture deconvolution

Differences in cellular composition across samples represent a potential confounder when testing associations between DNA methylation and quantitative traits in EWAS [17]. Cellular proportions for each sample can be estimated through cytometric methods or by applying cell mixture deconvolution algorithms to DNA methylation measurements [18, 19]. Cellular proportions can then be incorporated into a statistical model as covariates to adjust for potential cellular heterogeneity. In the absence of direct cell counts or tissue-specific reference DNA methylomes, statistical methods that account for cell proportion variability across tissue samples without a reference DNA methylome have been widely used [18, 20–23]. To perform a reference-free EWAS we used the R package RefFreeEWAS to deconvolute the cellular populations present in the tissue biopsy samples using DNA methylation data as detailed previously in Houseman et al. [23]. Briefly, this method seeks to represent the largest axes of variation in the DNA methylation data set and decomposes the DNA methylation data for a sample of heterogeneous cell populations into its constituent methylomes. As a convex variant of non-negative matrix factorization, the RefFreeEWAS method is similar to approaches used to deconvolute gene expression levels in heterogeneous tumor tissues [24, 25]. In the present study, we selected the 10,000 most variable CpGs in each data set and used a bootstrap technique (specifically sampled the specimens with replacement 1000 times) to estimate the optimal number of putative cell types (K). The optimal number of cell-types defined in each data set was: K = 6 (Komen), K = 10 (TCGA adjacent normal), and K = 2 (NDRI normal breast). The discrepancy in estimated cell-types for each population can be explained in part by the sample size (i.e., small for the NDRI population) and potential epigenomic field defects in normal-adjacent to tumor tissue (i.e., TCGA).

#### Analysis of CpG-specific associations

We used a multivariable linear models for microarray data (limma) procedure as described in the R/bioconductor library *limma* [26] to model CpG-specific associations between logit-transformed beta values (i.e., *M* values) and breast cancer risk factors (e.g., age, BMI, parity). Genome-wide significance was determined by taking into account the false discovery rate with a threshold of statistical significance set at *Q* = 0.01. We ran separate multivariate limma models both unadjusted and adjusted for putative cell proportions to assess the impact of cell proportion differences on significant associations and effect-size estimates. To identify loci that may be most confounded by differences in cell type we calculated the difference in the effect-size estimates (i.e., delta coefficient value) between the cell-type unadjusted and adjusted models.

#### Associations with metadata

To test the associations between putative cellular proportions and subject metadata (e.g., age) we applied the methods described in Houseman et al. to fit a quasi-binomial model for each putative cell-type across the data set [23]. More specifically, for each estimated value of K (that is, total number of cell types), we generated a model for each cell type (1 to K) and used the minimum *P* value. We then computed the permutation distribution of these minimum *P* values (spanning all potential values of K).

#### Genomic region enrichment

To assess the enrichment of risk-factor-related CpG sites at cell-type-specific histone modifications we used the eFORGEv1.2 tool with the selected option of all H3 marks measured for the consolidated Roadmap to Epigenomics data set [27]. To examine whether risk-factor-related CpGs were associated with transcription factor binding sites in ENCODE data we used the Locus Overlap Analysis (LOLA) software [28]. In this analysis, our query input set of genomic regions to be tested for enrichment were the genomic locations of the risk-factor-related CpG sites (*Q* < 0.01) and the background set was the genomic locations of the 390,262 CpGs used in the entire analysis. For the LOLA, the ENCODE transcription factor binding sites included 42 different chromatin immunoprecipitation sequencing experiments.

#### Epigenetic clock analysis

DNA methylation age (biological age) of the Komen breast tissues was calculated using the Horvath and epigenetic timer of cancer (EpiTOC) methods [29, 30].

## Results

### Differential DNA methylation is associated with breast cancer risk factors in normal breast tissues

Patient demographics and characteristics are presented in Table 1. The study participants ranged in age from 18 to 82 years with a median age of 37. A small proportion of participants were underweight (2%; BMI <18), 40% in the normal BMI range (> = 18 and <25), 30% were overweight (> = 25 and <30), and 28% were obese (>30). Over half of subjects had at least one full-term birth (56%), and the remaining 44% were nulliparous. To test the hypothesis that DNA methylation differences in normal breast tissue are related to known breast cancer risk factors we used the approach outlined in Additional file 1. Using the RefFreeEWAS deconvolution algorithm, we identified the optimal number of putative cell-types as K = 6 as this estimate minimized the deviance of the bootstraps (see “Methods” and Additional file 2A). To investigate whether the heterogeneity in cellular proportions across samples was associated with phenotypic variables (e.g., subject age) we applied a quasi-binomial model for each subject. To avoid dependence on the selection of K (putative cell-types) we examined associations over a range of evaluated K using a permutation test (1000 permutations) for inference of each phenotypic variable. As shown in Additional file 2B, estimated cell mixture proportions were significantly associated with subject age (permutation *P* value = 2.0E-03), but not subject BMI or parity (Additional file 2B).

**Table 1:** Subject demographics and characteristics

Variable	Value in subjects (*n* = 100)
Age (median, range)	37.2 (18–82)
Body mass index (median, range)	27.6 (16.8–53.7)
Pregnancy (parity), *n*	
No	44
Yes	56
Family history, *n*	
No	44
Yes	46
Missing	10
Race, *n*	
African American	5
Hispanic	9
White	86
Alcohol consumption - drinks per week, *n*	
Not current drinker	28
<7	64
7–14	5
15–21	2

**Fig. 1:**
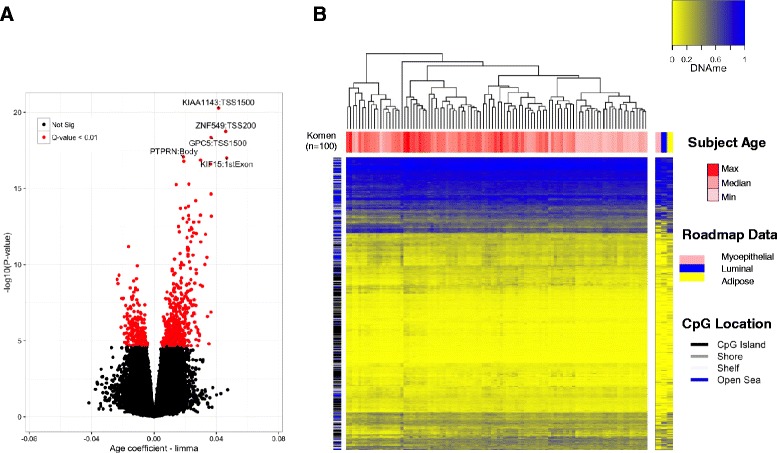
Subject age is strongly associated with DNA methylation in normal breast tissue independent of cell type. **a** In the volcano plot, each point represents the associations between DNA methylation and age from cell-type-adjusted multivariable linear models for microarray data (limma) at individual cytosine-guanine dinucleotide (CpG) sites. Increasing -log10 (*P* value) values on the *y-axis* show increasing statistical significance and limma effect size on the *x-axis* positioned away from the *zero value* reveal the largest DNA methylation changes with age. Significant CpG sites are indicated in *red* (*Q* value < 0.01). The gene and gene regions are presented for the five CpG sites with the greatest significance. **b** Unsupervised clustering of DNA methylation values at age-related CpG sites (Komen, *n* = 100) visualized alongside CpGs measured in specific cell-types form the Roadmap to Epigenomics data set (*n* = 691 CpG sites). Each *column* represents a given tissue sample and each CpG is presented in rows

To study the relationship between DNA methylation and breast cancer risk factors we applied both unadjusted and cell-type-adjusted linear models for microarray (limma) to examine the influence of subject age, BMI, and parity on the DNA methylome. Since the estimated cellular proportions for each sample sum to nearly one, we included all but the estimated cell-type with the smallest proportion to avoid multi-collinearity in our models. In a multivariable limma model adjusted for differences in cellular mixtures, 787 CpG sites were significantly associated with age, 0 CpG sites were associated with BMI, and 0 CpG sites were associated with parity, after correcting for multiple hypothesis testing (*Q <* 0.01, Fig. 1a). The full list of 787 CpG sites with genome annotation and statistical results is presented in Additional file 3. Notably, age-related DNA methylation alterations were predominantly hypermethylation events, i.e., increased DNA methylation was associated with increased age (545 CpG sites, 69.3%). To assess the impact that adjusting for cellular proportions had on the identification of significant associations and effect sizes, we computed the difference between the coefficients (i.e., a delta coefficient value) at each CpG for the models unadjusted and adjusted for cell type. A large CpG-specific delta value provides evidence for associations between DNA methylation and risk factors that may be most confounded by differences in cellular proportions. Visualization of CpG-specific *P* values and coefficients from cell-type unadjusted and adjusted models demonstrated that adjustment attenuated both the strength and magnitude of CpG-specific associations genome-wide (Additional file 4). Moreover, the number of significant associations (*Q* < 0.01) in the unadjusted limma model for subject age was 4099 CpG sites compared with 787 from the adjusted model, suggesting that a large number of false-positives are likely to be reported when differences in cell proportions are not considered (Additional file 4A-C). In addition, at the age-related CpG sites (*n* = 787, *Q* < 0.01) the DNA methylation patterns across purified cell populations of myoepthial cells, luminal cells, and adipocytes were consistent, suggesting that age-related changes may occur largely independent of tissue type in the normal human breast (Fig. 1b).

There were missing data on family history in 10 individuals in the present data set. To explore whether family history was associated with DNA methylation differences we applied the aforementioned limma approach unadjusted and adjusted for cellular proportions (*n* = 90), and found no significant associations (*Q* > 0.01) between family history and DNA methylation differences after correcting for multiple comparisons (Additional file 4D).

### Independent validation of age-associated methylation

We next moved to validate our age-related DNA methylation findings in two independent 450 K data sets from 97 normal adjacent-to-tumor breast samples (TCGA) and 18 normal breast tissues from disease-free women (NDRI, GSE74214). Subject demographics and characteristics for these two data sets are presented in Table 2. In a reference-free cell-mixture-adjusted limma restricted to the 787 CpG sites identified in the discovery (Komen) population we observed that 548 CpG sites (TCGA, 69.4%) were differentially methylated in a direction consistent with the discovery population at a nominal *P* value <0.05 (Additional file 5A). Similarly, we observed highly consistent results in the NDRI population (389 out of 787 CpG sites, 49.4%) (Additional file 5B). Strikingly, there were 345 CpG sites (43.8%) in the TCGA data set and 109 CpGs (13.9%) in the smaller NDRI data set that were considered significant at the stringent Bonferroni threshold for multiple comparisons (Additional file 5A and B, *P* < 6.4E-05). In both validation cohorts, putative cell-mixture proportions were significantly associated with subject age (permutation *P* < 0.05) (Additional files 5C-D).

**Table 2:** Independent population subject characteristics

NDRI		
	Mean (range)	*n* = 18
Age	49 (13–80)	
BMI	28.3 (14.59–62.73)	
TCGA		
	Mean (range)	*n* = 97
Age	57.57 (28 − 90)	
BMI	Unavailable	

*NDR*I National Disease Research Interchange, *TCGA* The Cancer Genome Atlas, *BMI* body mass index

While it is appreciated that DNA methylation can modify chromatin structure and distally regulate the transcriptome, the most well-defined function of DNA methylation is the *cis*-regulation of gene transcription [31]. In the present study, sample-matched RNA-sequencing data were available only for a subset of the subjects from the TCGA data set (*n* = 88). Many of the age-related CpG sites that localize to gene regions (*n* = 630 CpG sites) demonstrated strong associations with gene expression (259 CpG sites at *P* < 0.05, Additional file 6A). The direction of the CpG-gene correlations demonstrated a dependency upon genomic context (Additional file 6B). For example, CpG sites tended to be negatively correlated in the promoter region, while there was an even distribution of positive and negative correlation in the gene body (that is, intron and exon) regions (Additional file 6B).

### Age-associated DNA methylation sites are enriched for regulatory regions

**Fig. 2:**
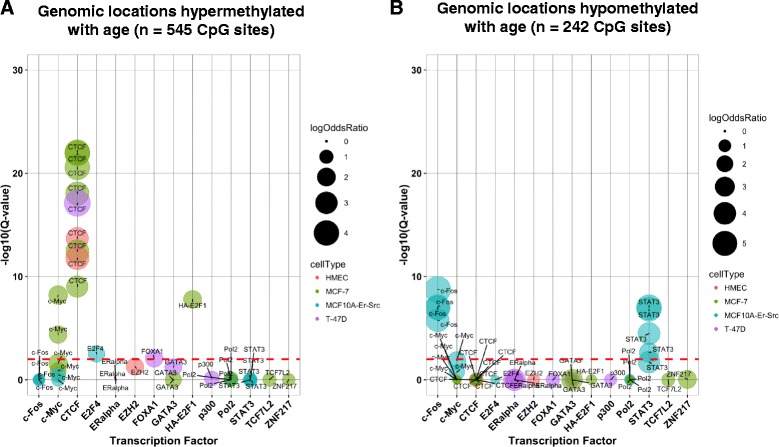
Age-related DNA methylation is enriched for regions of chromatin remodeling and transcriptional control. Cytosine-guanine dinucleotide (*CpG*) sites hypermethylated with age (**a**) and CpG sites hypomethylated with age (**b**) are highly enriched at the binding sites of transcription factors

To provide a broader biological interpretation of age-related DNA methylation we next sought to identify enrichment of these genomic locations in gene regulatory regions, such as tissue-specific histone marks and transcription factor binding sites (TFBS). First, we employed the eFORGE tool to identify cell-type-specific signals in diverse tissues profiled by the Roadmap to Epigenomics Consortium. We observed robust enrichment of H3K4me1, histone modifications that mark enhancers, in both fetal tissues and mammary epithelial cells (*Q* < 1.9E-37), and modest associations with other histone modifications (i.e., H3K4me3, H3K27me3) (Additional file 7). Fisher’s exact test confirmed that age-related CpGs localize to enhancer elements specifically in mammary myoepithelial cells (H3K4me1, Roadmap) (OR = 2.00 CI (1.73–2.33), *P =* 7.1E-20). We next used the genomic coordinates of age-related CpGs as a query set against the background of the 450 K array in LOLA scanning for enrichments of TFBSs. Since hypermethylation events are likely to be biologically distinct from hypomethylation events at TFBS we stratified our LOLA into a hypermethylation and a hypomethylation enrichment analysis (Fig. 2a and b). In the hypermethylation analysis, we observed a striking number of significant enrichments for CpG sites that were hypermethylated with age (14 TFBS, *Q* < 0.01) and hypomethylated with age (8 TFBS, *Q* < 0.01) (Additional file 8A and B). Among several of the top-ranking results presented in Fig. 2a, MYC and CTCF, which are critical regulators of chromatin architecture were enriched among hypermethylated CpG sites, while hypomethylated CpGs localize to binding sites of transcriptional activators c-Fos and Stat-3 [32–35].

### Accelerated epigenetic aging of human breast tissue

It has been recognized that DNA methylation patterns change in a tissue-specific manner as an individual ages [29]. Previous studies have found that measurements of DNA methylation have the ability to accurately estimate an individual’s age and that observed differences between predicted DNA methylation age (that is, biological age) and chronological age are associated with disease-risk factors [29, 30, 36, 37]. Further, it has been observed that DNA methylation age predictions in the human breast demonstrate age acceleration when compared with other tissues, suggesting that normal breast tissue tends to age more quickly than other tissues [29].

**Fig. 3:**
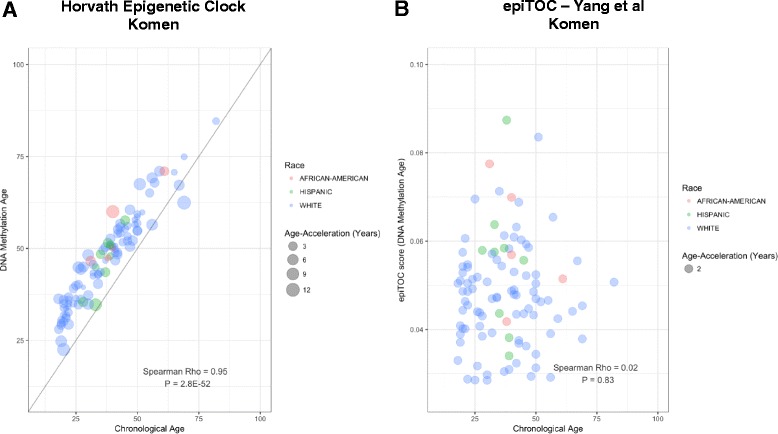
Relationship between epigenetic clocks and cancer risk factors. **a** The Horvath epigenetic clock age in normal breast tissue is highly correlated with subject age (*P* = 2.83E-52). Age acceleration was significantly (*P* < 0.05) larger in African American women. **b** DNA methylation age as generated by the epigenetic timer of cancer (epiTOC) tool was not correlated with subject age in normal breast tissue (*P* > 0.05). Higher DNA methylation age was associated with subject race, as breast tissue from African American and Hispanic women demonstrated increased DNA methylation age (*P* < 0.05)

To examine whether the subject-specific differences between biological and chronological age (that is, age acceleration) are associated with breast cancer risk factors we first calculated DNA methylation age from the 100 Komen normal breast tissue samples using two distinct epigenetic clocks [29, 30]. Briefly, the “Horvath epigenetic clock” uses elastic net regression to integrate DNA methylation information from 353 CpG sites to generate a multi-tissue age predictor. The second method, “epiTOC”, is an epigenetic clock that incorporates prior biological knowledge into a mathematical model to generate an estimate of mitotic divisions using 385 CpG sites. Notably, there was limited overlap between the 787 age-related CpGs and Horvath (17 CpGs) and EpiTOC (3 CpGs). In analyses with the Horvath clock, we observed strong positive correlation between chronological age and the DNA methylation age of the Komen breast tissues, with a Spearman correlation coefficient of 0.95 (*P* = 2.83E-52, Fig. 3a). In univariate analyses of age acceleration, defined as the residual resulting from regressing DNA methylation age (Horvath clock) on chronological age, and the cancer risk factors listed in Table 1, we observed a significant positive association only with race (African American, *n* = 5 subjects, *P* = 3.5E-02). Age acceleration was not associated with any other of the evaluated risk factors (*P* > 0.05). In a multivariate model considering all measured cancer risk factors, we found that race was significantly associated with increased epigenetic aging (African American *P* = 4.9E-02). In contrast to the Horvath clock, there was no significant correlation between chronological age and epiTOC-predicted age (*P* = 7.5E-01, Fig. 3b). Nonetheless, the epiTOC estimated biological age was also positively associated with race in univariate analyses (African American *P* = 2.1E-02, Hispanic *P* = 2.8E-02) and in multivariate models including all risk factors shown in Table 1 (African American *P* = 2.7E-02, Hispanic *P* = 2.7E-02). The remaining breast cancer risk factors were not associated with epiTOC-defined biological aging in either univariate or multivariate models (*P* > 0.05).

### Age-related DNA methylation is further deregulated in pre-invasive and invasive breast cancer

**Fig. 4:**
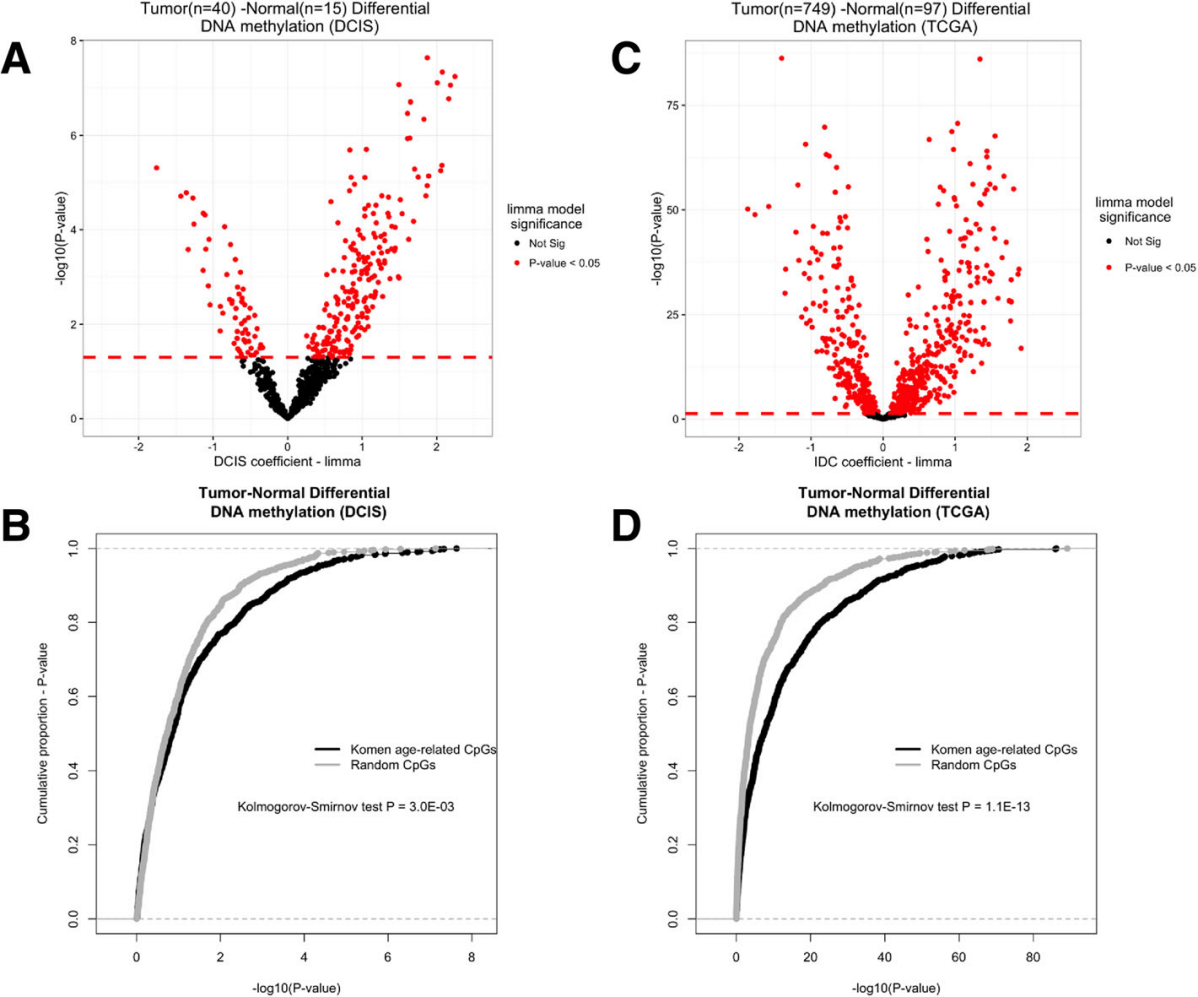
DNA methylation differences between tumor and normal breast tissue at age-related cytosine-guanine dinucleotide (*CpG*) sites in both ductal carcinoma in situ (*DCIS*) (**a**, **b**) and invasive breast cancer (**c**, **d**)

To ascertain whether differences in DNA methylation in relation to disease risk factors are relevant for the development of cancer, we compared DNA methylation in breast tumors with adjacent normal tissue in both pre-invasive and invasive cancer, at the 787 age-related CpGs. In pre-invasive lesions (ductal carcinoma in situ, DCIS), there were 268 CpG sites among 775 CpGs available for measure (34.5%) that demonstrated differential methylation between DCIS and normal tissue using limma models adjusted for subject age Fig. 4a (*P* < 0.05). Importantly, changes at the age-related CpGs were greater (Additional file 9A and B) and demonstrated stronger associations than a randomly selected set of CpG sites with similar properties regarding their location within CpG islands Fig. 4b (Kolmogorov-Smirnov test, *P =* 3.0E-03). If the epigenetic defects in age-related DNA methylation are further deregulated in pre-invasive breast cancer it would be expected that progressive changes would occur in invasive breast cancer. To test this, we assessed differential methylation using limma models adjusted for subject age in TCGA breast cancer data set. A large proportion of the age-related CpGs exhibited significant differential DNA methylation changes in breast cancer (642 out of 787 CpGs (81.6%, *P* < 0.05)) (Fig. 4c). Again, we found that the age-related changes demonstrated greater DNA methylation differences (Additional file 9C and D) and stronger associations than a randomly selected set of CpGs with matching genomic distribution (Kolmogorov-Smirnov test, *P =* 1.1E-13) (Fig. 4d).

## Discussion

In this study, we identified perturbations in the normal breast epigenome that may contribute to age-related increases in breast cancer risk. Age is the strongest demographic risk factor for breast cancer and is robustly associated with DNA methylation changes. Emerging literature has demonstrated that aging exerts its profound effects on the epigenome through a lifetime accumulation of environmental exposures that interfere with the placement or removal of methyl groups [11, 12, 38, 39]. Here, we have described that the consistent changes in breast DNA methylation are not randomly distributed throughout the genome. Instead, age-related DNA hypermethylation events are enriched for breast epithelial-specific enhancer regions and the binding sites of chromatin remodelers, while hypomethylation was noted at transcriptional activators. The enrichment of modifications at critical regulators of cellular phenotype provide novel insights into how cell-type-specific epigenetic states change over time and may predispose cells to neoplastic transformation. Our analysis revealed that further DNA methylation alterations to these genomic regions in pre-invasive and invasive disease may contribute to the restriction of cellular differentiation and disruption of transcriptional control observed in cancerous lesions.

The ability to produce reliable biological age predictions in an individual and in specific tissues holds promise for monitoring health, predicting disease risk, and providing insights into modifiable lifestyle factors that promote healthy aging. Indeed, discrepancies between chronological and biological age may suggest deregulation in DNA methylation marks and indicate increased disease risk. Horvath et al. demonstrated this phenomenon of age acceleration in a recent publication, whereby researchers found that the epigenetic age of the liver was increased by 2.7 years for every 10 units of BMI [36]. Using 450 K methylation arrays we have applied the Horvath epigenetic clock algorithm and epiTOC tool to 100 normal breast tissue samples to determine the DNA methylation age of each of these tissues. While there was an association between age acceleration and race in the Komen dataset, there were only five African American women, and this association requires additional analyses. Profiling a larger number of breast tissue samples from African-American women would allow investigation of whether genetic differences are associated with accelerated aging. In future studies, the ability to accurately assess biological age, in breast tissue samples from larger longitudinal studies with a greater number of women of diverse racial and ethnic backgrounds, may aid researchers in the determination of factors that aim to assess and prevent disease.

While our findings provide strong evidence for a link between epigenetic deregulation and the two processes of aging and cancer, our study had a few limitations. For example, the questionnaire administered by the Susan G. Komen Tissue Bank is well-equipped to accurately classify a subject’s age, while other cancer risk factors such as average alcohol consumption may be impacted by recall bias. This same limitation applies to the missing data on family history in 10 subjects. Further, the small number of non-white subjects decreased the power to identify relationships between age acceleration and race. Separately, although the RefFreeEWAS method effectively accounts for the largest sources of variation in the DNA methylation data set, the method is unable to discern the particular cell types in which epigenetic changes occur. That said, the robustness of cell-type-independent observation across multiple populations and progressive alterations in cancer gives us confidence that a subset of the epigenetic defects may be important in carcinogenesis. To this end, future prospective studies are needed to investigate the relationship between DNA methylation in normal tissue and the risk of developing breast cancer. Finally, mechanistic studies will also be needed to elucidate the epigenetic contribution to increased breast cancer risk. Research aimed at early detection and disease prevention would serve to relieve patient morbidity and reduce the cost burden to the healthcare system.

In summary, we have shown that epigenetic differences are strongly associated with aging and these differences may reflect epigenetic defects that predispose women at an older age to an increased risk of breast cancer.

## Conclusions

Epidemiological studies have firmly established factors of personal choice and factors beyond personal choice that alter the risk of breast cancer. Established risk factors for breast cancer include age, reproductive and family history, and BMI [4, 40]. Indeed, modeled breast cancer risk factors have been shown to account for approximately half of breast cancer cases [41, 42]. However, the biological mechanisms by which specific risk factors impact disease risk are not well-understood. In this study population, we did not observe significant associations between BMI or parity and genome-wide DNA methylation. However, we observed consistent cell-type-independent age-related DNA methylation in normal breast tissue from multiple populations. The genomic locations of age-related DNA methylation were more likely to be found in gene regulatory elements of breast epithelial cells, suggesting a loss of cellular state control as an individual ages. Further, we demonstrated additional support for a link between age-related DNA methylation and cancer, as age-related CpG sites were more likely to exhibit greater alterations in both pre-invasive and invasive breast cancer. Together, our research suggests that DNA methylation changes in aging shift the epigenetic state toward a compromised molecular phenotype, creating a novel link between the risk factor of age and the potential origins of disease in breast cancer.

## Additional files

Additional file 1: Analytic framework for reference-free epigenome-wide association study between DNA methylation and breast cancer risk factors. (PPTX 77 kb)

Additional file 2: Estimation of cellular proportions and its association with subject covariates. **A** Hierarchal clustering and heat map representation of cellular proportions of putative cell-types (K = 6) in Komen normal breast tissue (*n* = 100). **B** Metadata associations with cellular proportions when K is estimated over a range of cell types. Permutation *P* values presented adjacent to the *colored line* representing each covariate (e.g., *red* for age, permutation *P* = 2.0E-03). (PPTX 424 kb)

Additional file 3: Age-related CpGs and genomic annotation. (XLSX 169 kb)

Additional file 4: Volcano plots representing both cell-type proportions adjusted and unadjusted limma models for each covariate evaluated in the present study. In each cell-type-adjusted volcano plot (*right panels*) the intensity of *blue* and *red points* indicate shift in the effect size of the limma coefficient estimate between adjusted and unadjusted models. That is, *gray points* in the *right panels* indicate CpG sites that are not impacted by differences in cellular proportions across subject age (n =100) (**A**), subject BMI (*n* = 100) (**B**), parity status (*n* =100) (**C**), and family history of disease (*n* = 90) **(D**). (PPTX 1367 kb)

Additional file 5: Age-related DNA methylation in the human normal breast validated in adjacent-to-tumor normal breast from The Cancer Genome Atlas (*TCGA*) population (*n* = 97) (**A**), and normal breast tissue from the National Disease Research Interchange (*NDRI*) population (*n* = 18) (**B**). Volcano plots indicate CpG-specific associations between DNA methylation and subject age. Permutation testing of subject covariate data across estimated cell-types (K) in the TCGA population (**C**) and NDRI population (**D**). (PPTX 923 kb)

Additional file 6: Age-related CpG sites are associated with gene transcription. **A** Distribution of *P* values for CpG-gene expression correlations. **B** Genomic-context dependency between DNA methylation and gene expression. Gene names for the 20 CpG-gene regions with the strongest associations are presented alongside its respective coefficient-*P*-value bubble. (PPTX 758 kb)

Additional file 7: Complete results from eFORGE analysis of age-related CpGs (*n* = 787). (XLSX 136 kb)

Additional file 8: Complete results from LOLA analysis of age-related CpGs (*n* = 787). (XLSX 71 kb)

Additional file 9: **A**, **B** DNA methylation differences between DCIS and normal adjacent tissue in limma coefficients (i.e., effect size) for age-related (*n* =787) and randomly selected loci (*n* = 787). **C**, **D** DNA methylation differences between invasive breast cancer and normal adjacent tissue in limma coefficients (i.e., effect size) for age-related (*n* = 787) and randomly selected loci (*n* = 787). (PPTX 970 kb)

## Abbreviations

5mC: 5-methylcytosine
BMI: Body mass index
CpG: Cytosine-guanine dinucleotide
DCIS: Ductal carcinoma in situ
ENCODE: Encyclopedia of DNA Elements
epiTOC: Epigenetic timer of cancer
EWAS: Epigenome-wide association study
GEO: Gene Expression Omnibus
IDAT: Intensity data file
limma: Linear models for microarray data
LOLA: Locus Overlap Analysis
NDRI: National Disease Research Interchange
PCGT: Polycomb group protein target
RefFreeEWAS: Reference-free DNA methylation mixture deconvolution epigenome-wide association study
TCGA: The Cancer Genome Atlas
TFBS: Transcription factor binding site
TSS: Transcriptional start site
