# Supplementary material for: Normal breast tissue DNA methylation differences at regulatory elements are associated with the cancer risk factor age
Source: Breast Cancer Res. 2017 Jul 10;19:81. doi: 10.1186/s13058-017-0873-y (PMC5504720; doi:10.1186/s13058-017-0873-y)
Supplement: Supplementary file 1 — Analytic framework for reference-free epigenome-wide association study between DNA methylation and breast cancer risk factors. (PPTX 77 kb) [file 13058_2017_873_MOESM1_ESM.pptx]

## Slide 1
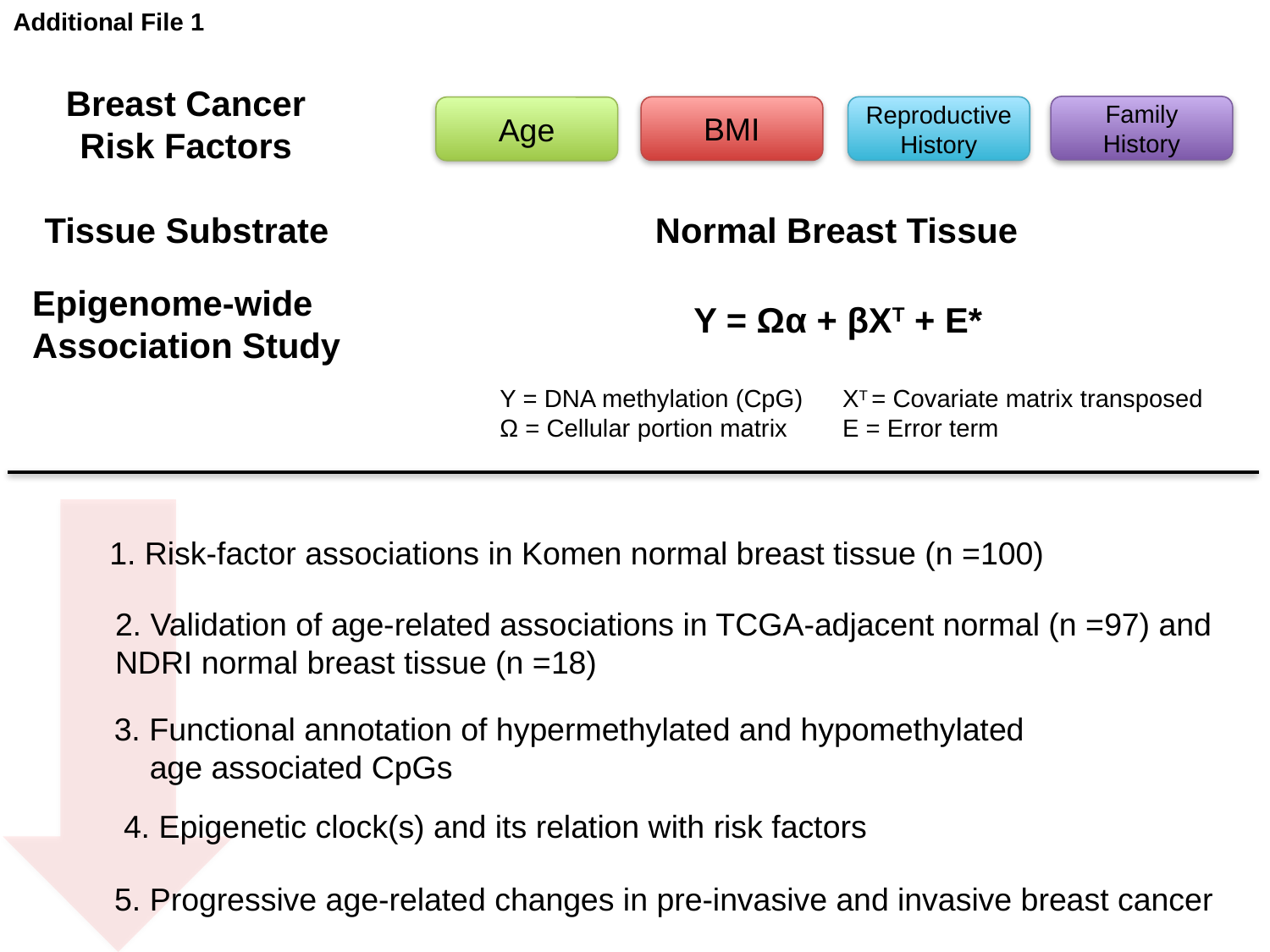

Additional File 1
Breast Cancer
Risk Factors
Family History
BMI
Reproductive History
Age
Tissue Substrate
Normal Breast Tissue
Epigenome-wide
Association Study
Y = Ωα + βXT + E*
Y = DNA methylation (CpG)
Ω = Cellular portion matrix
XT = Covariate matrix transposed
E = Error term
1. Risk-factor associations in Komen normal breast tissue (n =100)
2. Validation of age-related associations in TCGA-adjacent normal (n =97) and
NDRI normal breast tissue (n =18)
3. Functional annotation of hypermethylated and hypomethylated
 age associated CpGs
4. Epigenetic clock(s) and its relation with risk factors
5. Progressive age-related changes in pre-invasive and invasive breast cancer
