# Supplementary figures and images for: Normal breast tissue DNA methylation differences at regulatory elements are associated with the cancer risk factor age
Source: Breast Cancer Res. 2017 Jul 10;19:81. doi: 10.1186/s13058-017-0873-y (PMC5504720; doi:10.1186/s13058-017-0873-y)

## Slide 1
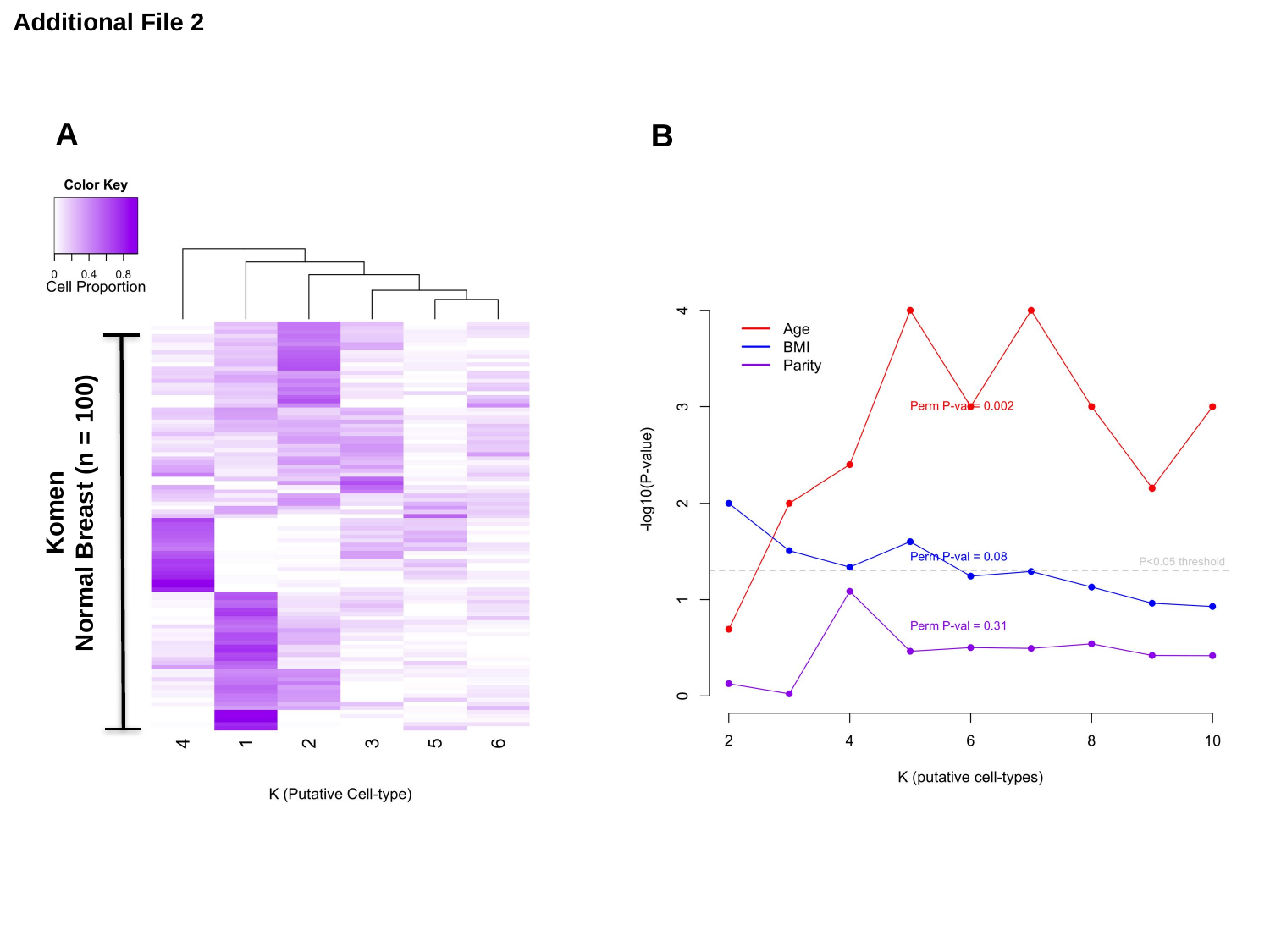

Additional File 2
A
B
Komen
Normal Breast (n = 100)

Supplement: Supplementary file 2 — Estimation of cellular proportions and its association with subject covariates. A Hierarchal clustering and heat map representation of cellular proportions of putative cell-types (K = 6) in Komen normal breast tissue (n = 100). B Metadata associations with cellular proportions when K is estimated over a range of cell types. Permutation P values presented adjacent to the colored line representing each covariate (e.g., red for age, permutation P = 2.0E-03). (PPTX 424 kb) [file 13058_2017_873_MOESM2_ESM.pptx]

## Slide 1
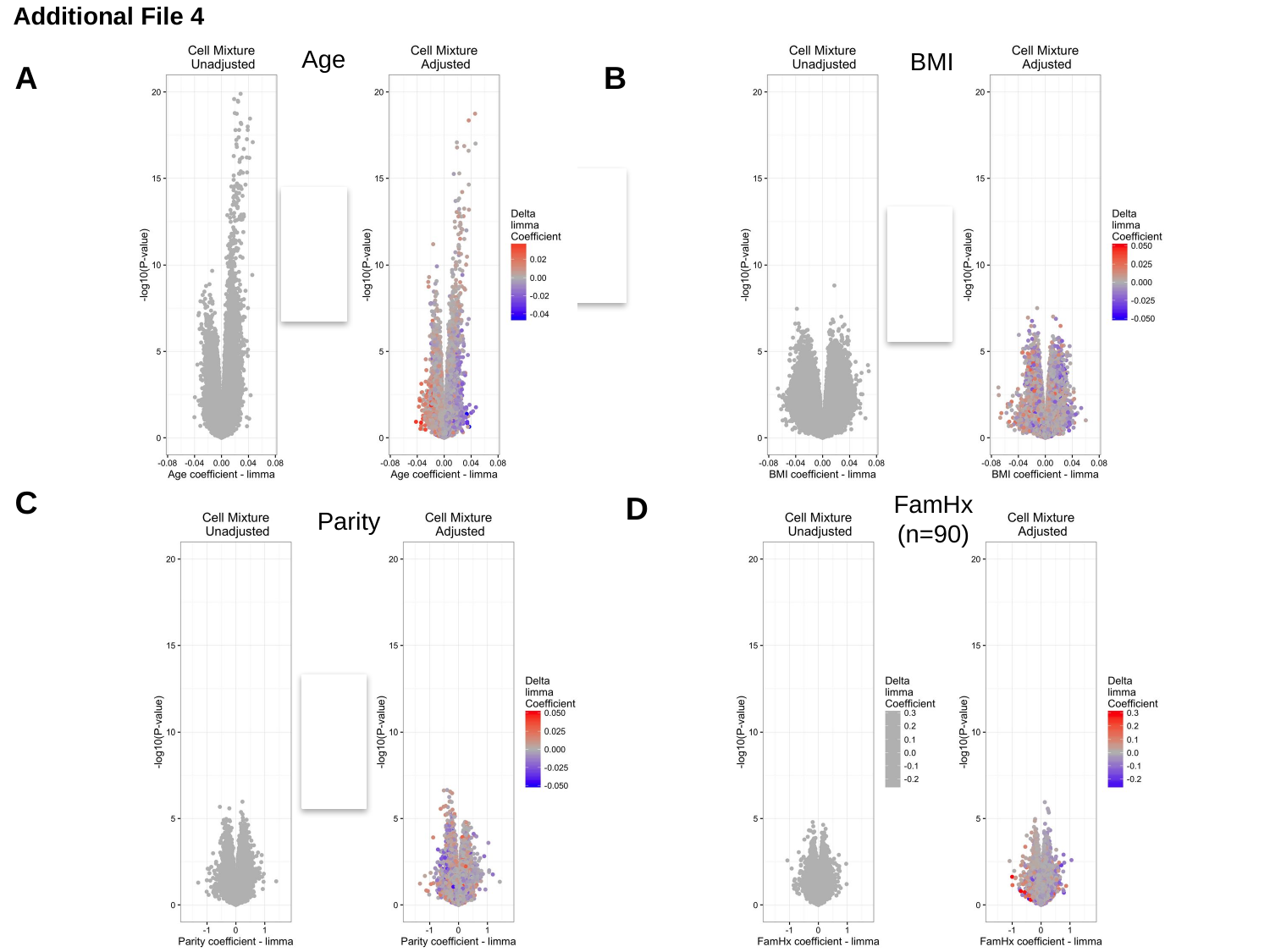

Additional File 4
Age
BMI
A
B
C
D
FamHx
(n=90)
Parity

Supplement: Supplementary file 4 — Volcano plots representing both cell-type proportions adjusted and unadjusted limma models for each covariate evaluated in the present study. In each cell-type-adjusted volcano plot (right panels) the intensity of blue and red points indicate shift in the effect size of the limma coefficient estimate between adjusted and unadjusted models. That is, gray points in the right panels indicate CpG sites that are not impacted by differences in cellular proportions across subject age (n =100) (A), subject BMI (n = 100) (B), parity status (n =100) (C), and family history of disease (n = 90) (D). (PPTX 1367 kb) [file 13058_2017_873_MOESM4_ESM.pptx]

## Slide 1
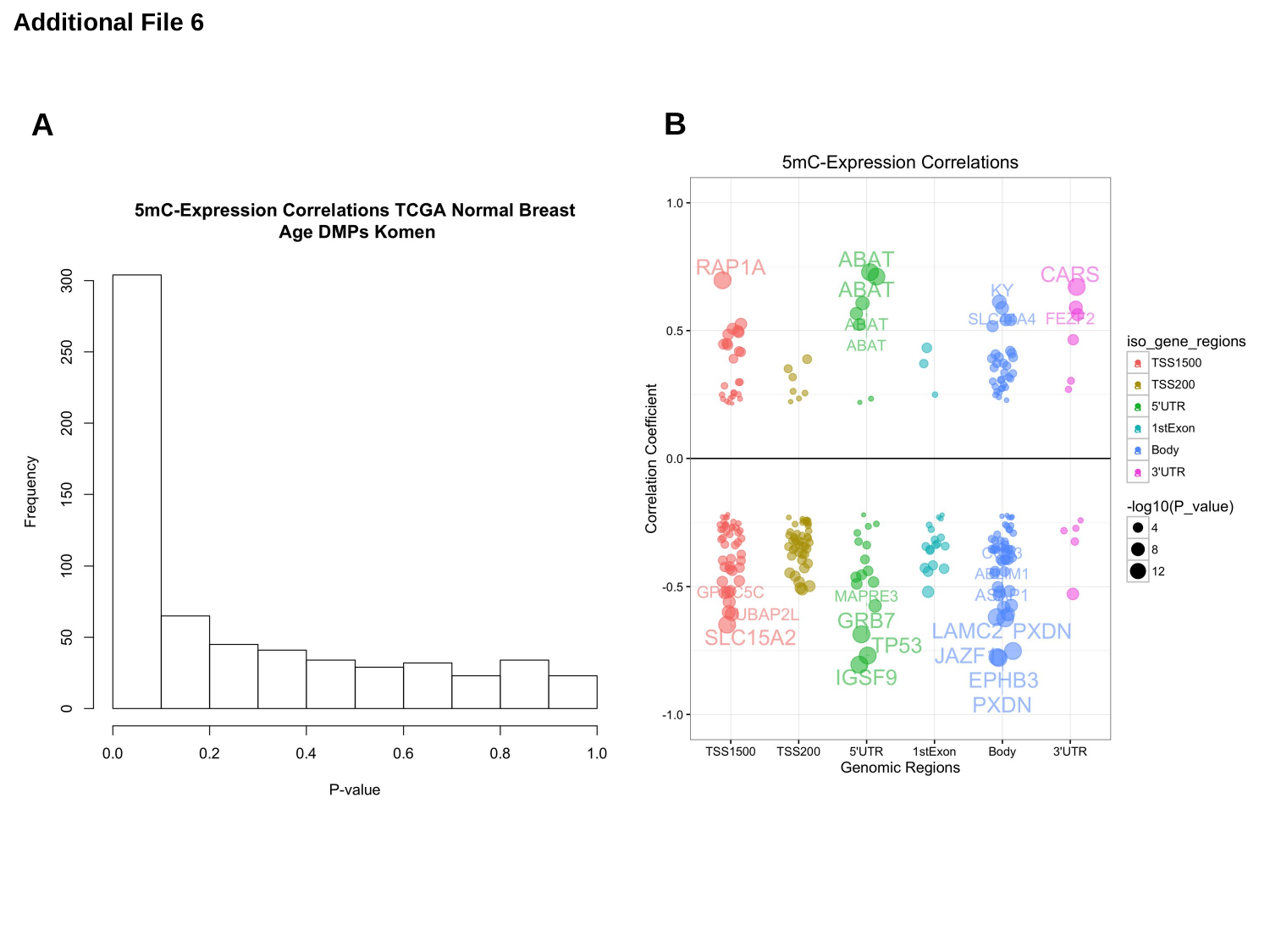

Additional File 6
B
A

Supplement: Supplementary file 6 — Age-related CpG sites are associated with gene transcription. A Distribution of P values for CpG-gene expression correlations. B Genomic-context dependency between DNA methylation and gene expression. Gene names for the 20 CpG-gene regions with the strongest associations are presented alongside its respective coefficient-P-value bubble. (PPTX 758 kb) [file 13058_2017_873_MOESM6_ESM.pptx]

## Slide 1
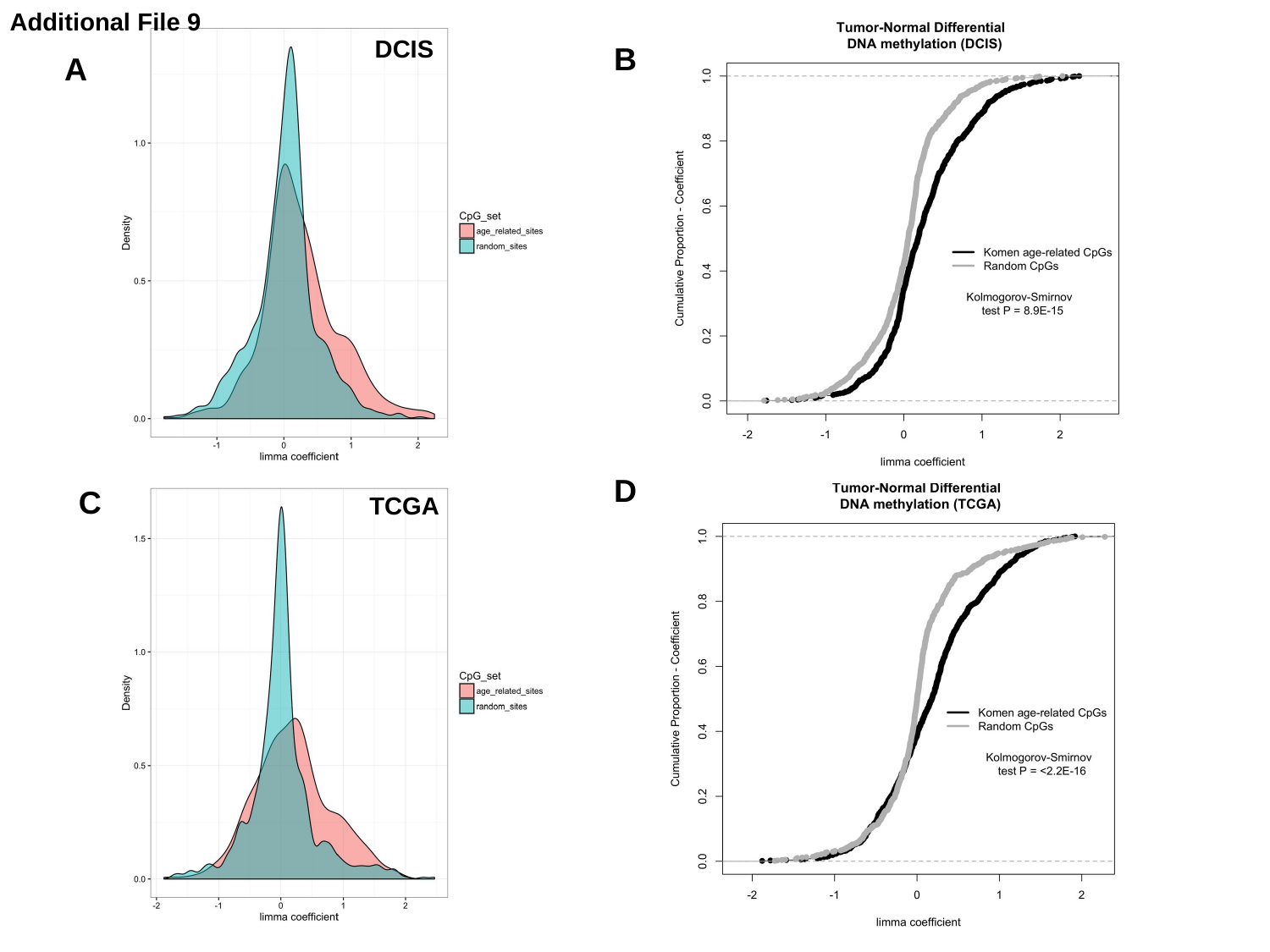

Additional File 9
DCIS
B
A
D
C
TCGA

Supplement: Supplementary file 9 — A, B DNA methylation differences between DCIS and normal adjacent tissue in limma coefficients (i.e., effect size) for age-related (n =787) and randomly selected loci (n = 787). C, D DNA methylation differences between invasive breast cancer and normal adjacent tissue in limma coefficients (i.e., effect size) for age-related (n = 787) and randomly selected loci (n = 787). (PPTX 970 kb) [file 13058_2017_873_MOESM9_ESM.pptx]
