# Supplementary material for: Normal breast tissue DNA methylation differences at regulatory elements are associated with the cancer risk factor age
Source: Breast Cancer Res. 2017 Jul 10;19:81. doi: 10.1186/s13058-017-0873-y (PMC5504720; doi:10.1186/s13058-017-0873-y)
Supplement: Supplementary file 5 — Age-related DNA methylation in the human normal breast validated in adjacent-to-tumor normal breast from The Cancer Genome Atlas (TCGA) population (n = 97) (A), and normal breast tissue from the National Disease Research Interchange (NDRI) population (n = 18) (B). Volcano plots indicate CpG-specific associations between DNA methylation and subject age. Permutation testing of subject covariate data across estimated cell-types (K) in the TCGA population (C) and NDRI population (D). (PPTX 923 kb) [file 13058_2017_873_MOESM5_ESM.pptx]

## Slide 1
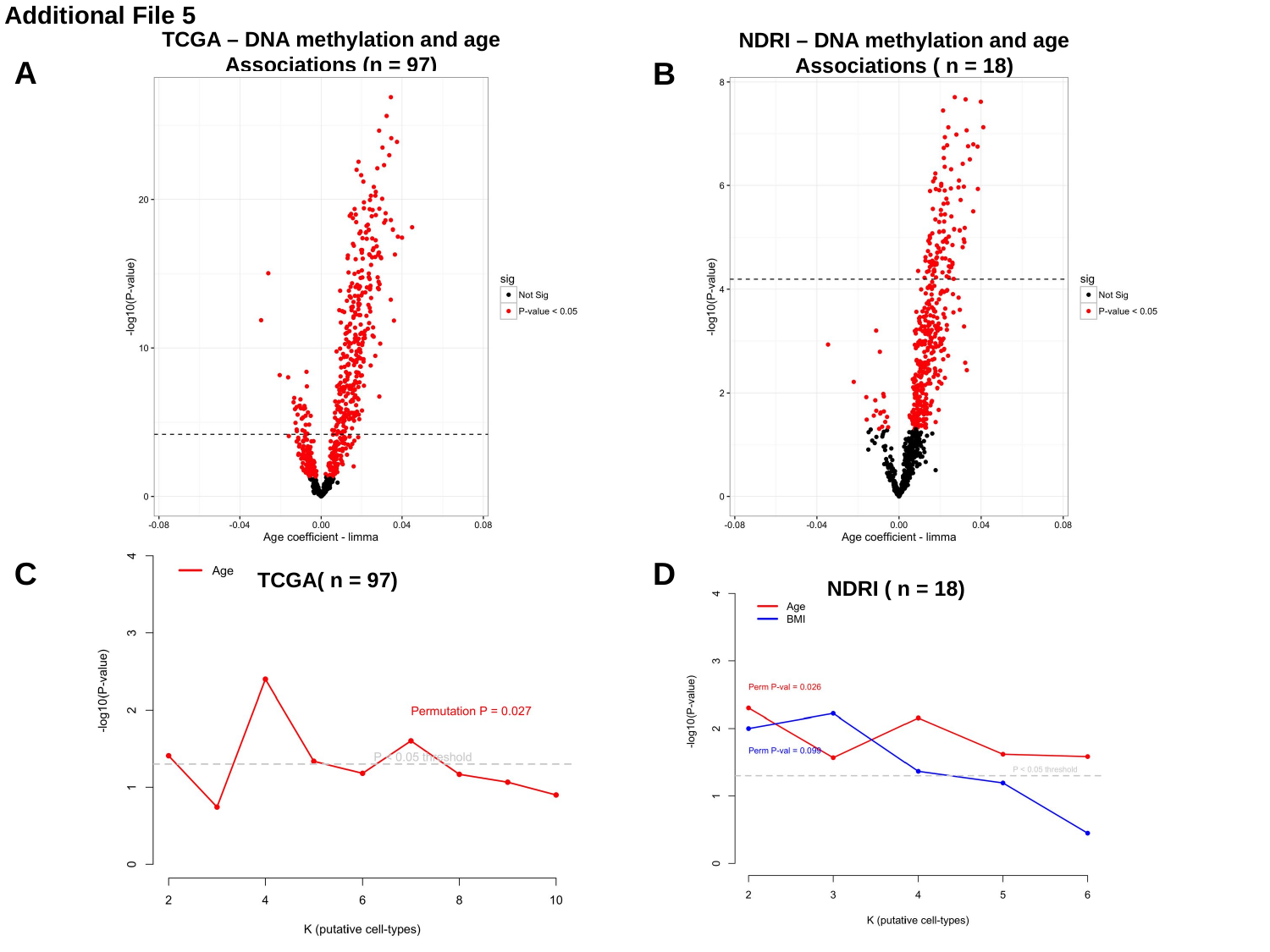

Additional File 5
TCGA – DNA methylation and age
Associations (n = 97)
NDRI – DNA methylation and age
Associations ( n = 18)
A
B
C
D
TCGA( n = 97)
NDRI ( n = 18)
